# The Pyrazolyl-Urea Gege3 Inhibits the Activity of ANXA1 in the Angiogenesis Induced by the Pancreatic Cancer Derived EVs

**DOI:** 10.3390/biom11121758

**Published:** 2021-11-24

**Authors:** Raffaella Belvedere, Elva Morretta, Nunzia Novizio, Silvana Morello, Olga Bruno, Chiara Brullo, Antonello Petrella

**Affiliations:** 1 Department of Pharmacy, University of Salerno, Viale Giovanni Paolo II, Fisciano, 84084 Salerno, Italy; rbelvedere@unisa.it (R.B.); emorretta@unisa.it (E.M.); nnovizio@unisa.it (N.N.); smorello@unisa.it (S.M.); 2 Department of Pharmacy, University of Genova, Viale Benedetto XV 3, 16132 Genova, Italy; obruno@unige.it (O.B.); brullo@difar.unige.it (C.B.)

**Keywords:** Gege3, annexin A1, extracellular vesicles, angiogenesis, PKCα inhibition, calcium mobilization, pancreatic cancer

## Abstract

The pyrazolyl-urea Gege3 molecule has shown interesting antiangiogenic effects in the tumor contest. Here, we have studied the role of this compound as interfering with endothelial cells activation in response to the paracrine effects of annexin A1 (ANXA1), known to be involved in promoting tumor progression. ANXA1 has been analyzed in the extracellular environment once secreted through microvesicles (EVs) by pancreatic cancer (PC) cells. Particularly, Gege3 has been able to notably prevent the effects of Ac2-26, the ANXA1 mimetic peptide, and of PC-derived EVs on endothelial cells motility, angiogenesis, and calcium release. Furthermore, this compound also inhibited the translocation of ANXA1 to the plasma membrane, otherwise induced by the same ANXA1-dependent extracellular stimuli. Moreover, these effects have been mediated by the indirect inhibition of protein kinase Cα (PKCα), which generally promotes the phosphorylation of ANXA1 on serine 27. Indeed, by the subtraction of intracellular calcium levels, the pathway triggered by PKCα underwent a strong inhibition leading to the following impediment to the ANXA1 localization at the plasma membrane, as revealed by confocal and cytofluorimetry analysis. Thus, Gege3 appeared an attractive molecule able to prevent the paracrine effects of PC cells deriving ANXA1 in the tumor microenvironment.

## 1. Introduction

It is known that the initiation of tumor angiogenesis is required for tumor progression, arousing a considerable interest from the research community stimulating extensive efforts based on an anti-angiogenic therapy to treat cancer [1]. During this pathological process, pro-angiogenic factors are initially secreted into the extracellular fluid to activate endothelial cells, which form a functional vascular network [2]. Indeed, among these factors, the tumor microenvironment consists of VEGF (Vascular Endothelial Growth Factor), bFGF (basic Fibroblast Growth Factor), and PDGF (Platelet-Derived Growth Factor), cytokines, small non-coding RNAs which strongly acts to promote angiogenesis mainly in a hypoxic environment [3].

In this scenario, the protein Annexin A1 (ANXA1) has been identified as a potential biomarker for tumor differentiation and prognosis, maintaining a tissue-specific role as an oncogene or oncosuppressor [4]. Its abnormal regulation (compared to a normal organization) may play an important role in the pathogenesis of cancer acting on cell proliferation, apoptosis, migration/invasion, and metastatization [5]. Moreover, the effects of ANXA1 in cancer possibly depend on its differential distribution among the cytoplasm, the nucleus, and the cell surface [6]. Regarding these changes in protein localization, it is known that the post-translational modifications of ANXA1, such as phosphorylation, are able to regulate its release in the extracellular environment [7]. Indeed, previous studies have revealed that in pancreatic cancer (PC) ANXA1 acts as an intra- and extracellular component promoting the progression. In particular, in the extracellular environment, ANXA1 is able to promote the sustainment and development of PC cells as a key player of extracellular vesicles (EVs), mainly exosomes. It has been shown that EVs deriving from PC cells are able to activate fibroblasts and endothelial cells in a paracrine manner through the interaction with the Formyl Peptide Receptors (FPRs), known to be ANXA1 receptor partner [8,9,10]. Interestingly, a direct link between the autocrine function of ANXA1 contained in EVs secreted by endothelial cells and the VEGF release has been described [11]. 

The block of the pro-angiogenic factors in tumor therapy is one of the major strategies in the treatment and still represents a challenge, mostly aimed at the inhibition of VEGF effects [12]. Among the chemical approaches to identify compounds able to selectively block the VEGF pathway, a recent chemical study has focused on the synthesis of molecules based on pyrazoles and imidazopyrazoles nuclei. By this research, a library of substances capable to decrease the VEGF-mediated phosphorylation levels of p38MAPK, ERK1/2, and Akt has been identified [13]. Furthermore, from this library the ethyl 1-(2-hydroxypentyl)-5-(3-(3-(trifluoromethyl) phenyl) ureido)-1H-pyrazole-4-carboxylate, named Gege3, has emerged as a promising anti-angiogenic compound, inhibiting endothelial tube formation by indirectly inhibiting intracellular calcium release [14,15].

Here, we showed the indirect link between the molecule Gege3 and the protein ANXA1 in the HUVEC cell line. In particular, the significant decrease of calcium intracellular level induced by Gege3, prevented the translocation to the plasma membrane of ANXA1, and strongly prevented the positive paracrine feedback on tumor-associated angiogenesis induced by Ac2-26 and PC deriving EVs, as both mediators of ANXA1 extracellular form. 

## 2. Materials and Methods

### 2.1. Cell Culture and Reagents

HUVEC cell line (Human Umbilical Vein Endothelial Cells) (ATCC^®^ PCS-100-010™; Manassas, VA, USA) was cultured as reported in [16]. MIA PaCa-2 cells (ATCC^®^ CRL-1420; Manassas, VA, USA) were grown as described in [17]. The ANXA1 KO stable clone from the MIA PaCa-2 cell line had been obtained as previously reported [17] through the CRISPR- Cas9 genome editing technology. Cells were stained at 37 °C in a 5% CO_2_−95% air humidified atmosphere. 

The ethyl 1-(2-hydroxypentyl)-5-(3-(3-(trifluoromethyl) phenyl) ureido)-1*H*-pyrazole-4-carboxylate (named Gege3 as in [13,14]) emerged from a library of pyrazolyl-ureas and imidazo-pyrazoles previously synthesized as described in [13]. Gege3 was dissolved in DMSO and diluted in the culture medium at the specified concentrations. VEGF-165 (VEGF; R&D Systems; Minneapolis, MN, USA) was suspended in sterilized and bi-distilled water at an initial concentration of 100 µg/mL. The stock solution of phorbol-12-myristate-13-acetate (PMA; Sigma Aldrich, St. Louis, MO, USA) and of Gö6976 [2-(2-Cyanoethyl)-6,7,12,13-tetrahydro-13-methyl-5-oxo-5H-indolo(2,3-a)pyrrolo (3,4-c)-carbazole] (Selleckchem; Houston, TX, USA) were prepared in DMSO at a concentration of 20 mM and 100 mM, respectively. Ac2-26 peptide (10 mM) (Tocris Bioscience, Bristol, UK) were suspended in phosphate saline buffer (PBS: 137 mM NaCl, 2.7 mM KCl, 10 mM Na_2_HPO_4_, 2 mM KH_2_PO_4_, pH 7.4). 

### 2.2. Exosomes Isolation

The extracellular vesicles (EVs) enriched in exosomes were obtained from MIA PaCa-2 and ANXA1 KO MIA PaCa-2 (confluence of about 8 × 10^7^ cells in 1.5 × 10^5^ cm^−2^, cultured for 24 h in DMEM without FBS) cell supernatants, after abundant washing with PBS. This medium was collected and processed according to the protocol used in [9]. In detail, the medium was centrifuged for 5 min at 300× *g* at room temperature (RT) to remove detached cells; the supernatant was transferred and centrifuged for 10 min at 2000× *g* at 4 °C to remove dead cells. The obtained supernatant was transferred and centrifuged at 10,000× *g* for 30 min at 4 °C to eliminate cell debris. Then, the cleared supernatant was transferred to ultracentrifuge tubes and centrifuged for 70 min at 100,000× *g* at 4 °C. The obtained pellet was washed in PBS and re-ultracentrifuged at 100,000× *g* at 4 °C for 70 min. Finally, the supernatant was removed and the pellet, composed of microvesicles, mainly exosomes, was resuspended. The buffer we selected for the resuspension was 200 µL of sterile PBS for the administration to cells and 50 µL of RIPA lysis buffer for Western blotting. The quantization was performed through the Bradford assay as reported in [10] using the bovine serum albumin (BSA) solubilized in RIPA buffer as a reference for a standard curve. This step has been important in order to know that the amount of MIA PaCa-2 EVs administered to cells and also analyzed through Western blotting was 20 μg.

### 2.3. Wound-Healing Assay

The confluent monolayer of HUVEC was scraped with a pipette tip to produce a wound. Next, the cells were treated according to the experimental points previously administered of mitomycin C (10 μg/mL, Sigma-Aldrich; St. Louis, MO, USA) to ensure the block of mitosis. The wounds were photographed and analyzed as reported in [18]. 

### 2.4. Invasion Assay

The invasion capability of cells was performed through the transwell systems (12 mm diameter, 8.0-fim pore size, Corning Incorporated, New York, NY, USA), as previously described [19]. The established treatment was added in the lower chambers of each well in experimental points, previously addition of mitomycin C (10 μg/mL, Sig-ma-Aldrich; St. Louis, MO, USA) to arrest mitosis. Staining and analysis procedures were reported in [20].

### 2.5. Tube Formation Assay 

The in vitro angiogenesis has been performed as reported in [21]. After 12 h, the 10x images were acquired by EVOS^®^ optical microscope (Life Technologies Corporation, Carlsbad, CA, USA) and analyzed both for length and the number of branches by the ImageJ software (NIH, Bethesda, MD, USA) (Angiogenesis Analyzer tool for ImageJ).

### 2.6. Measurement of Intracellular Ca^2+^ Signaling

Intracellular Ca^2+^ concentrations [Ca^2+^] were measured using the fluorescent Fluo-4 a.m. probe (Molecular Probes, Thermo Fisher Scientific, Waltham, MA, USA), as previously described [22]. Briefly, HUVEC cells were trypsinized, washed, and placed in 1.5 mL tubes at 5 × 10^5^/mL and then incubated with Gege3 (10 μM final concentration) in PBS 1× for 1 h at RT. Then, cells were washed by centrifugation (5 min at 300× *g*) and incubated with 2.5 μM of Fluo-4 a.m. in 3% DMSO (DMSO final concentration in each assay was 0.06%) at 37 °C for 45 min in PBS 1×. Finally, cells were re-washed and the fluorescence in each sample was analyzed by a BD FACSCalibur cytometer (Becton Dickinson FACScan, Franklin Lakes, NJ, USA) using the 530/30 filter. Before reading, cells were treated with VEGF (50 ng/mL), Ac2-26 (1 μM), and EVs (20 μg). Rapid kinetic measurement of fluorescence was performed by flow cytometry using Ca^2+^-ionophore (ionomycin 1 mM; Sigma-Aldrich, St. Louis, MO, USA) and the chelating agent EDTA (15 mM, 15 min before; Sigma-Aldrich, St. Louis, MO, USA) as the positive and negative controls, respectively.

### 2.7. Confocal Microscopy 

HUVEC cells seeded on glass-bottom in the multiwell plate were fixed in p-formaldehyde at 4% *v*/*v* in PBS, (Lonza; Basel, Switzerland), permeabilized with Triton X-100 at 0.5% *v*/*v* in PBS (Lonza; Basel, Switzerland) and then blocked with goat serum at 20% *v*/*v* in PBS (Lonza; Basel, Switzerland). Next, incubation with the antibody against ANXA1 (rabbit polyclonal, 1:100; Thermo Fisher Scientific; Waltham, MA, USA), overnight at 4 °C was performed. After two washing steps, cells were incubated with anti-rabbit AlexaFluor 555 (1:500; Thermo Fisher Scientific; Waltham, MA, USA) for 2 h at room temperature (RT) in the dark. To detect nuclei DAPI (1:1000) was used. Samples were vertically scanned through the Leica SP8 confocal microscope (Leica Microsystems CMS Gmbh; Mannheim, Germany). The calculation of ANXA1 fluorescence signal on the HUVEC plasma membrane has been calculated as CMCF (Corrected Membrane Cell Fluorescence) by using Image J (NIH, Bethesda, MD, USA). By this software, we first have subtracted the extracellular black signal as background for all calculations. Next, the CMCF has been calculated by subtracting the cytosol fluorescent signal from the membrane one whose profile has been created by following cell perimeter. Both cytosol and membrane signals have been captured as the mean of at least five points randomly selected within cell area and perimeter, respectively. These analyses have been performed on at least ten cells for each image. The number values obtained have been inserted in Excel and the percentage and the statistical analysis have been performed. 

### 2.8. Flow Cytometry

HUVEC cells, differently treated, were harvested at a number of 5 × 10^5^/mL, and the pellets were incubated for 1 h at RT in PBS 1x containing FBS (2% *v*/*v*), used to avoid non-specific interactions, and anti-human antibody against ANXA1 (rabbit polyclonal, 1:500; Thermo Fisher Scientific; Waltham, MA, USA) and then for another hour with anti-rabbit 555-conjugated secondary antibody (1:500; Thermo Fisher Scientific; Waltham, MA, USA). Finally, the expression of the cellular marker was analyzed by flow cytometer (Becton Dickinson FACScan, Franklin Lakes, NJ, USA) using the Cells Quest program, by which the quantification analysis of positive events on dot plots have been performed.

### 2.9. HUVEC Cytosol and Membrane Protein Extracts

Cytosol and membrane proteins have been separately obtained by using the Plasma Membrane Protein Extraction Kit (Abcam, Cambridge, UK). After treatments, 1 × 10^6^ HUVEC cells for each experimental point were washed twice with PBS, detached with trypsin-EDTA 1× in PBS, harvested in PBS, and centrifuged for 5 min at 600× *g* at 4 °C. After that, cells were manually lysed in 2 mL homogenized buffer mix with a Dounce homogenizer, alternating lysis cycles (5 min) with pause ones (2 min), three times. All these steps were performed on ice. The obtained samples were then centrifuged at 4 °C for 10 min, at 700× *g*. The resulting supernatants were centrifuged again for 30 min at 10,000× *g* at 4 °C, until new supernatants were obtained corresponding to cytosol extracts. Each resultant pellet, equivalent to the total membrane protein content, was dissolved in RIPA buffer as reported above.

### 2.10. Western Blotting 

Proteins extracted from cells were examined by SDS-PAGE. Protein content was estimated according to Bradford assay (BIO-RAD, Hercules, CA, USA), as previously described [23]. We have analyzed primary antibodies against pPKCα (phospho-Protein Kinase C alpha; rabbit polyclonal; 1:500; Thermo Fisher Scientific, Waltham, MA, USA), ERK (rabbit monoclonal; 1:1000; Cell Signaling Technology, Danvers, MA, USA), pERK (rabbit monoclonal; 1:1000; (phospho-Thr202/Tyr204; Cell Signaling Technology, Danvers, MA, USA), calreticulin (rabbit polyclonal; 1:1000; Elabscience; Houston, TX, USA), TSG101 (tumor susceptibility gene 101; mouse monoclonal; 1:1000; ThermoFisher Scientific; Waltham, MA, USA), CD81 (mouse monoclonal; 1:500; BD Pharmingen, Franklin Lakes, NJ, USA), CD63 (mouse monoclonal; 1:500; BioLegend; San Diego, CA, USA); pSer27-ANXA1 (rabbit polyclonal; 1:500, homemade, see [24]), GAPDH (glyceraldehyde 3-phosphate dehydrogenase; mouse monoclonal; 1:1000; Santa Cruz Biotechnologies; Dallas, TX, USA) β-actin (mouse monoclonal; 1:1000; Santa Cruz Biotechnologies; Dallas, TX, USA). The blots were exposed to Las4000 (GE Healthcare Life Sciences; Little Chalfont, UK) and the relative band intensities were determined using ImageQuant software (GE Healthcare Life Sciences; Little Chalfont, UK).

### 2.11. Statistical Analysis

All the data and statistical analyses were made with Microsoft Excel. We reported the number of independent repetitions and p values in the legends of the figures for each experiment. All results are the mean ±standard deviation of at least 3 experiments performed in triplicate. The statistical data analysis was performed thanks to the two-tailed *t*-test comparing two variables and the differences were considered significant if *p* < 0.05, *p* < 0.01, and *p* < 0.001.

## 3. Results

### 3.1. Gege3 Inhibits the Pro-Angiogenic Effects of ANXA1 Mimetic Peptide, Ac2-26

**Figure 1:**
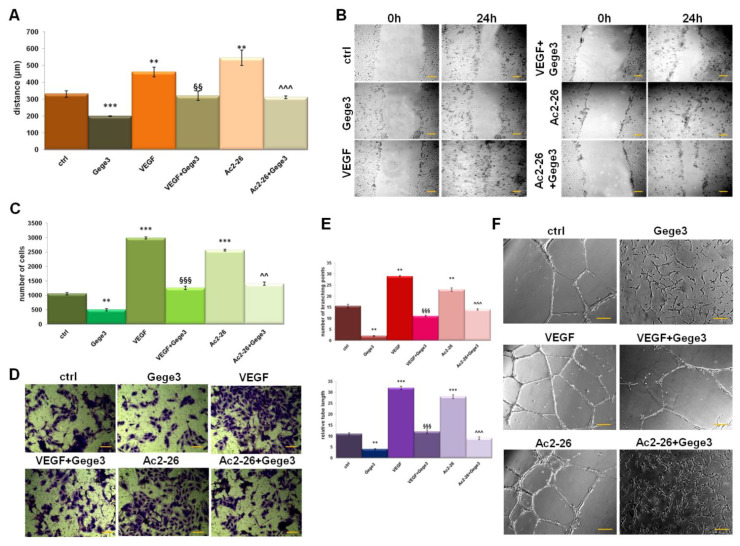
Effects of Gege3 on HUVEC cells. (**A**) Migration, (**C**) invasion speed, and (**E**) in vitro angiogenesis of HUVEC cells treated with VEGF 10 ng/mL, Ac2-26 1 µM, Gege3 10 µM. Each kind of evaluation has been accompanied by representative bright-field images ((**B**) for wound healing, (**D**) for invasion, and (**F**) for angiogenesis). The angiogenesis has been evaluated through the tube length and the number of branches calculated by ImageJ (Angiogenesis Analyzer tool) software. Histograms and representative bright-field images for each assay are reported. The bars of each bright-field image are 50 μm. Data represent the mean of four independent experiments ± standard deviation with similar results. ** *p* < 0.01; *** *p* < 0.001 for treated cells vs. untreated controls; §§ *p* < 0.01; §§§ *p* < 0.001 for the point with VEGF+Gege3 vs. VEGF; ^^ *p* < 0.01; ^^^ *p* < 0.001 for Ac2-26+Gege3 vs. Ac2-26.

The effects of the molecule Gege3 in interfering with the VEGF activity about HUVEC cells angiogenesis and migration have been previously reported [13,14]. In parallel, the notable pro-angiogenic effects of Ac2-26, as mimetic of ANXA1 protein, represent a well-known concept [25,26]. In this work, the migration and invasion processes on HUVEC cells have been investigated. Interestingly, we observed that Gege3 suppressed the activity of Ac2-26. Indeed, the migration and invasion speed of HUVEC cells, strongly enhanced by the ANXA1 mimetic peptide, returned to very similar levels to untreated cells (Figure 1A,B for wound healing assay and Figure 1C,D for and invasion one, respectively). We also found that VEGF, used as technical control, was significantly inhibited by Gege3. The same trend has been observed for in vitro angiogenesis which we have evaluated in terms of tubes length and number of branching points. Particularly, both in presence of VEGF and Ac2-26 the endothelial cells increased their ability to form capillary-like structures. Conversely, when at the same experimental points we have added the Gege3, the angiogenic process notably underwent a decrease (Figure 1E,F). All the analyses of migration and invasion rate and angiogenesis have been accompanied by bright-field representative images (Figure 1B,D,F). 

### 3.2. Gege3 Interferes with Calcium Mobilization and with the Translocation of ANXA1 to Plasma Membrane of HUVEC Cells

**Figure 2:**
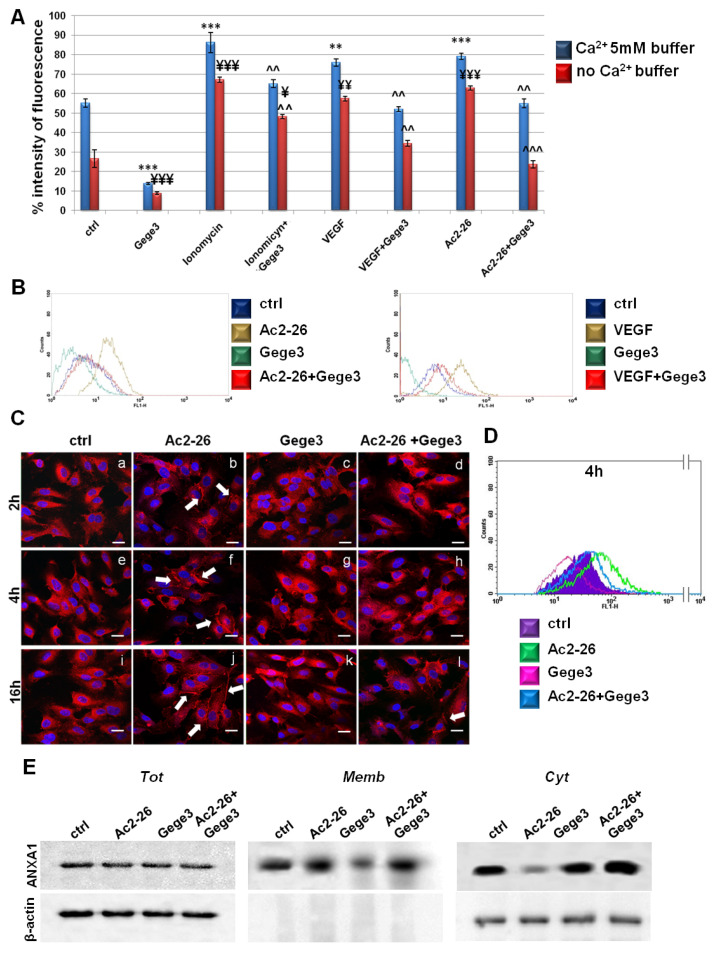
Analysis of Ca^2+^ mobilization and ANXA1 localization in HUVEC cells. (**A**) Effects of Gege3 10 μM, ionomycin 1 mM with and without Gege3, VEGF 10 ng/mL with and without Gege3, and Ac-2-26 1 μM with and without Gege3, all monitored using the probe Fluo-4 a.m. In the histogram blue bars refer to the cytometry analysis of cells in PBS 1× complemented with CaCl_2_ 5 mM, red ones are related to the same experimental points evaluated in PBS 1× without calcium. (**B**) Representative scatter plots histograms of the cytometry analysis by Fluo-4 a.m. assay. The experimental points of ctrl (blue line), Ac2-26 1 µM or VEGF 10 ng/mL (brown line), and Gege3 10 µM alone (dark green line) and together (red line), in Ca^2+^ free PBS 1× are reported as indicated in the legend. (**C**) Immunofluorescence analysis to detect ANXA1 intracellular localization after 2 (panels **a**–**d**), 4 (panels **e**–**h**), and 16 (panels **i**–**l**) hours of treatments with Gege3 10 µM and Ac2-26 1 µM alone and together. Magnification 63 × 1.4 NA. Bar = 100 μm. (**D**) Cell surface expression of ANXA1 was analyzed by flow cytometry. The violet area in the plot is relative to not treated cells; the ANXA1 signal is shown in green after treatment with Ac2-26 1 µM, in pink with Gege3 10 µM, and in blue with both of them for 4 h. (**E**) Whole, membrane, and cytosol expression of ANXA1 in HUVEC cells treated or not for 8 h with Ac2-26 1 μM and Gege3 10 μM taken alone or together was analyzed by Western blot as described in the Materials and Methods section. Protein normalization and the check of the sample quality were performed on β-actin levels. Data are means ± standard deviation of three experiments with similar results. ** *p* < 0.01; *** *p* < 0.001 for treated cells vs. non treated control in buffer with Ca^2+^ 5 mM; ¥ *p* < 0.05; ¥¥ *p* < 0.01; ¥¥¥ *p* < 0.001 for treated cells vs. non treated control in buffer without Ca^2+^; ^^ *p* < 0.01; ^^^ *p* < 0.001 for treatments (ionomycin, VEGF and Ac2-26) with Gege3 vs. the same experimental points without Gege3, both with and without Ca^2+^.

Once we have found that the Ca^2+^ intracellular transition appeared inhibited by Gege3 in a concentration-dependent manner [14], we further proved that this molecule is able to suppress the effects of VEGF and Ac2-26 blocking Ca^2+^ mobilization. In particular, both in presence of external calcium and in absence of this one, the Fluo-4 a.m. cytofluorimetric assay highlighted that the growth factor and Ac2-26 strongly acted as inducers of calcium release, instead, in case of pretreatment with Gege3, the signal considerably decreased. This kind of effect has been found also for iomonycin that we have used as technical control (Figure 2A). Representative cytometry plots of HUVEC cells treated or not with Ac2-26 or VEGF and Gege3 together and alone in absence of external Ca^2+^ have been reported in Figure 2B. The evaluation of fluorescence intensity has been calculated on the same number of cells for each experimental point.

Next, we assessed the intracellular localization of the protein ANXA1 in HUVEC cells following Ac2-26 and Gege3 treatments, alone or together. In Figure 2B, through a confocal analysis, we have shown that the molecule of our interest inhibited the translocation to the plasma membrane of ANXA1 (Figure 2C, panels d, h, l) induced by its same mimetic peptide (Figure 2C, panels b, f, j, white arrows). Panels c, g, k show ANXA1 intracellular localization in presence of Gege3: the intracellular protein placement did not undergo a significant change, exhibiting a general diffuse signal. The ANXA1 fluorescence intensity at the different levels in endothelial cells has been also analyzed as described in the Materials and Methods sections and the results reported in Supplementary Figure S2A, further highlighted the increase of ANXA1 signal at the plasma membrane in presence of Ac2-26 and the following rescue when the peptide has been administered together with Gege3. The immunofluorescence results have been also confirmed by a cytofluorimetric evaluation at 4 h of treatments, which we have chosen as a representative experimental point. In this case, Figure 2D shows the signal of ANXA1 on plasma membrane for the untreated HUVEC cells in purple, in presence of Ac2-26 in green, after treatment with Gege3 in pink and with the co-administration of Ac2-26 and Gege3 in blue. Moreover, in Supplementary Figure S3A, it has been reported the quantitative analysis of the flow cytometry evaluation, confirming the results here described. 

In order to further support this result, western blotting has been performed on compartmentalized HUVEC protein extracts. As revealed in Figure 2E, the ANXA1 signal at plasma membrane becomes notably significant with Ac2-26, to consequentially decrease in the cytosol compartment of the same experimental point. The experimental time selected for the western blot analysis has been 8 h to include a further middle time in the whole evaluation. When also in presence of Gege3, the ANXA1 membrane signal appeared strongly reduced in opposition to the related cytosol one. In no case, the ANXA1 level change in total extracts. The densitometry analysis has been reported in Supplementary Figure S4, it has been performed on β-actin signals for total and cytosol ANXA1 ones and red ponceau for membrane extracts, panels A and B.

### 3.3. The Effects of MIA PaCa-2 EVs on ANXA1 Translocation to HUVEC Plasma Membrane Are Blocked by Gege3

**Figure 3:**
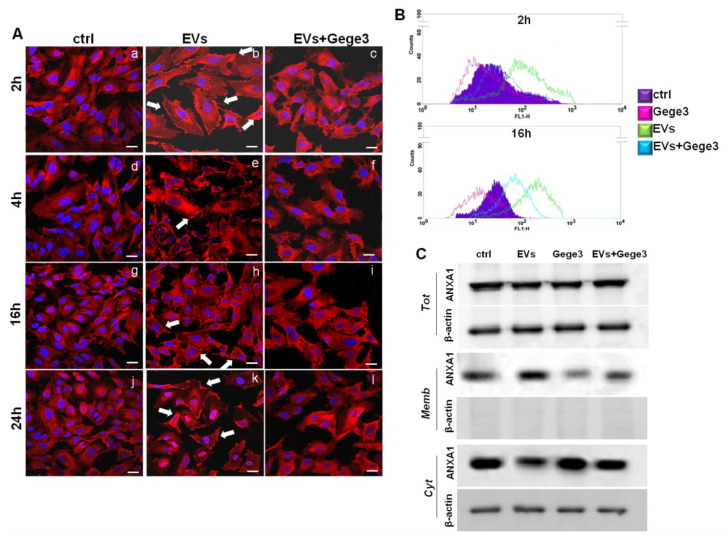
Evaluation of Gege3 effects on MIA PaCa-2 cells deriving EVs. (**A**) Confocal analysis for ANXA1 on HUVEC cells treated or not with EVs with and without Gege3 10 µM for 2 (panels **a**–**c**), 4 (panels **d**–**f**), 16 (panels **g**–**i**), and 24 (panels **j**–**l**) hours. Magnification 63 × 1.4 NA. Bar = 100 μm. (**B**) Cell surface expression of ANXA1 was analyzed by flow cytometry. The violet area in the plot is relative to not treated cells; the ANXA1 signal is shown in green after treatment with MIA PaCa-2 EVs, in pink with Gege3 10 µM, and in light blue with both of them for 2 and 16 h. (**C**) Whole, membrane, and cytosol expression of ANXA1 in HUVEC cells treated or not for 8 h with PC EVs and Gege3 10 μM taken alone or together, analyzed by Western blot. Protein normalization and the check of the sample quality were performed on β-actin levels.

The paracrine effects of MIA PaCa-2 cells derived from EVs have been described in-depth [9,10]. We first characterized the EVs by protein markers as TSG101, CD81, CD63 which are known to be present in the microvesicles, and by the absence of calreticulin as already described and here further shown in Supplementary Figure S1A [10,11]. The western blotting shown in this panel assessed the typical expression of ANXA1 in the total protein lysate of MIA PaCa-2 cells and the EVs harvested from these. Western blotting analysis showed for the first time, the phosphorylated form of ANXA1 on the residue of serine 27 in the EVs harvested from MIA PaCa-2 cells. This western blot also characterized the EVs, besides the total protein content, of ANXA1 KO MIA PaCa-2 cells, as a further in vitro PC model whose EVs have been used as reported below. In general, these microvesicles induced important activating effects on cell components of the tumor microenvironment which favor the PC progression. We have used the EVs secreted by MIA PaCa-2 PC cells as reported in the Material and Methods Section, to treat the HUVEC cell line which has been later analyzed through immunofluorescence assay. As reported above, the confocal analysis shown in Figure 3A (panels b, e, h, k, white arrows) has revealed that ANXA1 considerably moved to the plasma membrane in response to PC EV. However, when EVs have been administered together with Gege3, this effect appeared inhibited as shown by the absence of ANXA1 signal at the plasma membrane (Figure 3A, panels c, f, i, l). The analysis of immunofluorescence assay reported in Supplementary Figure S2B, further confirmed that EVs, only if administered alone, has been able to trigger the translocation of ANXA1 to the plasma membrane, instead of when together with Gege3, this process appeared not induced. These data have been confirmed through flow cytometry, at the two representative experimental times of 2 and 16 h, after treatments with EVs and Gege3 alone and together (Figure 3B). Additionally, the results obtained by this last technique have been further supported by the quantitative analysis on positive cell events for ANXA1 membrane staining, as reported in the histogram in Supplementary Figure S3B.

Moreover, the western blotting shown in Figure 3C has been further confirmed that ANXA1 translocated to the plasma membrane following the treatment for 8 h, chosen as intermediate time, with MIA PaCa-2 EVs and mainly that this process appeared inhibited by the co-administration of Gege3. The densitometry analysis has been included in Supplementary Figure S4C.

### 3.4. Gege3 Is Able to Inhibit Cell Motility and Angiogenesis Induced by PC Cells EVs

**Figure 4:**
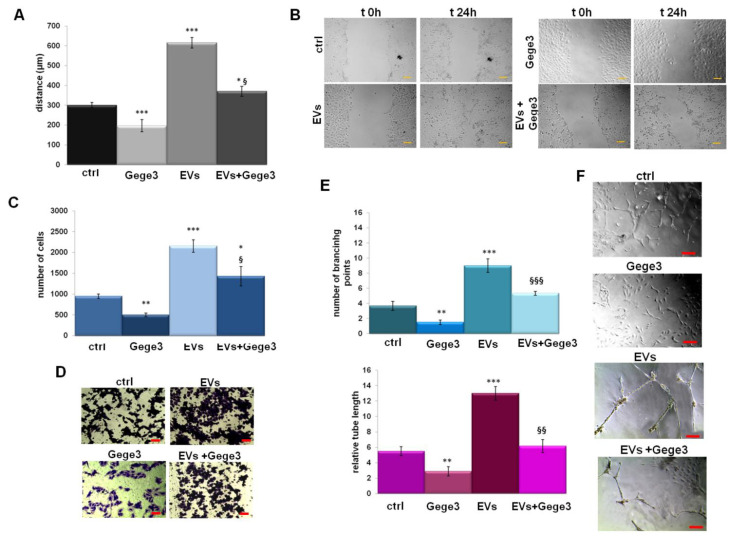
The influence of Gege3 on EV activity. (**A**) Migration, (**C**) invasion speed, and (**E**) in vitro angiogenesis of HUVEC cells treated with EVs secreted from MIA PaCa-2 cells in the presence or not of Gege3 10 µM. Representative bright-field images are reported for wound healing assay (**B**), for invasion one (**D**) and angiogenesis (**F**). The bars of each bright-field image are 50 μm. Data represent the mean of three independent experiments ± standard deviation with similar results. * *p <* 0.05; ** *p* < 0.01; *** *p* < 0.001 for treated cells vs. untreated controls; § *p <* 0.05; §§ *p* < 0.01; §§§ *p* < 0.001 for the point with EVs + Gege3 vs. EVs.

In order to analyze the role of Gege3 as an interfering molecule on EVs activity, we assessed the migration and invasion processes on HUVEC cells. In Figure 4A,B we showed the confirmation of positive effects of MIA PaCa-2 EVs in the induction of endothelial cells motility. The addition of Gege3 strongly reverted the EVs effects, so much that the migration and invasion speed of co-treated HUVEC cells returned to the control levels (Figure 4A,C for wound-healing and invasion assays with the related representative images in B for migration and D for invasion assays, respectively). The angiogenesis process has been further analyzed showing that, also, in this case, Gege3 notably inhibited the pro-angiogenic effects of PC EVs (Figure 4E). All the results here reported have been also highlighted by bright-field representative images (Figure 4A for migration assay, B for invasion one, F for in vitro angiogenesis).

### 3.5. The Effects of Gege3 Are Mediated by the Inhibition of PKCα

**Figure 5:**
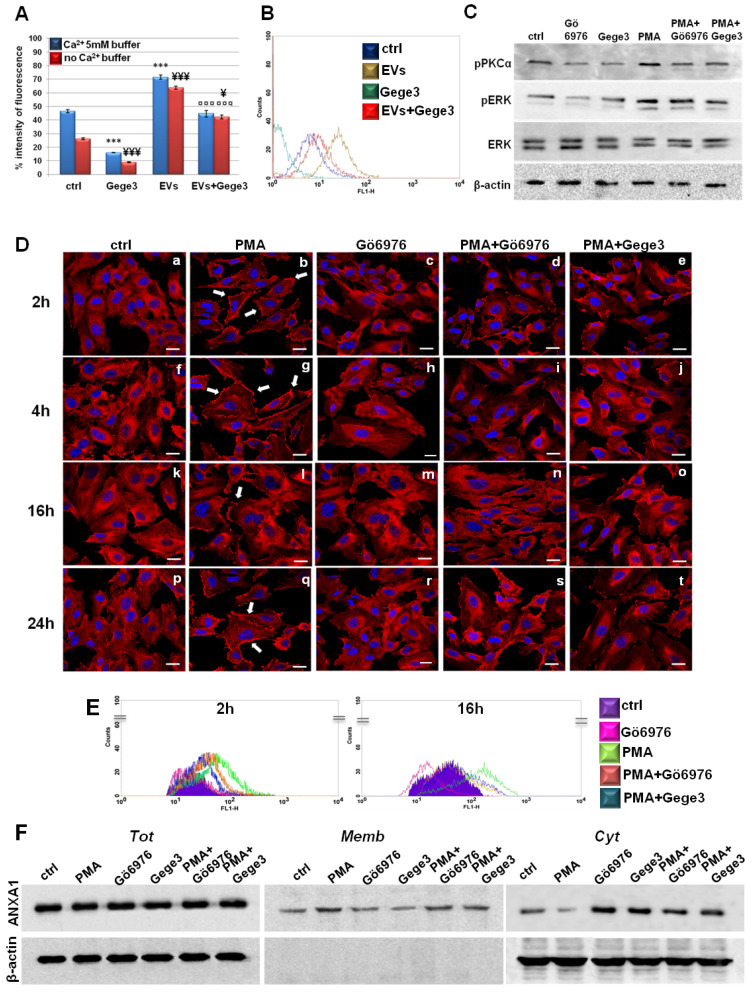
Endothelial cells answers to EVs in presence of Gege3 (**A**) Effects of Gege3 (10 μM), PC-derived EVs with and without Gege3, monitored using the probe Fluo-4 a.m. In the histogram blue bars refer to the cytometry analysis of cells in PBS 1× complemented with CaCl_2_ 5 mM, red ones are related to the same experimental points evaluated in PBS 1× without calcium. Data are means ± standard deviation of three experiments with similar results. *** *p* < 0.001 for treated cells vs. non treated control in buffer with Ca^2+^ 5 mM; ¥ *p* < 0.05; ¥¥¥ *p* < 0.001 for treated cells vs. non treated control in buffer without Ca^2+^; ¤¤¤ *p* < 0.001 for EVs+Gege3 vs. EVs alone both in the presence and absence of Ca^2+^. (**B**) Representative scatter plot histogram of the cytometry analysis by Fluo-4 a.m. assay. The experimental points of ctrl (blue line), EVs (brown line), and Gege3 10 µM alone (dark green line) and together (red line), in Ca^2+^ free buffer, are reported as indicated in the legend. (**C**) Western blotting for pPKCα, pERK, ERK, normalized with β-actin on HUVEC cells treated with PMA 100 nM, Gege3 10 µM, Gö6976 1 µM, PMA+Gö6976, PMA+Gege3 for 15 min. Cropped blots from full-length gels are representative of three independent experiments with similar results. (**D**) Immunofluorescence analysis to detect ANXA1 intracellular localization after 2 (panels **a**–**e**), 4 (panels **f**–**j**), 16 (panels **k**–**o**), and 24 (panels **p**–**t**) hours of treatments with PMA 100 nM, Gö6976 1 µM, PMA+Gö6976, PMA+Gege3. Magnification 63 × 1.4 NA. Bar = 100 μm. (**E**) Flow cytometry analysis for cell surface expression of ANXA1. The violet area in the plot is relative to not treated cells; the ANXA1 signal is shown in pink with Gö6976 1 µM, in green after treatment with PMA 100 nM, in orange in presence of PMA+Gö6976, and in blue with PMA+Gege3 for 2 and 16 h. (**F**) Whole, membrane, and cytosol expression of ANXA1 in HUVEC cells treated or not for 8 h with the same treatments described above. The data are representative of four independent experiments with similar results.

Similar to what has been found about the Ac2-26 effects, MIA PaCa-2 EVs alone and together with Gege3 have been administered to HUVEC cells to prove the variation of Ca^2+^ levels as reported in the Materials and Methods section. In Figure 5A we reported that the release of Ca^2+^ induced by EVs has been significantly reduced in presence of Gege3. The histogram shows that the same results have been obtained with and without the external addition of Ca^2+^. Additionally, we proved these effects through a representative scatter plot in Figure 5B of the Fluo-4 a.m. assay performed in the calcium-free buffer. In order to include further proof of the involvement of ANXA1 in the analyzed processes, we used EVs harvested from ANXA1 KO MIA PaCa-2 cells generated as reported in [17]. This stable in vitro model had already allowed us to show the importance of our protein of interest as an oncogenic factor in PC progression also through EVs effects [9,10,17]. Here, we showed in Supplementary Figure S5 that the ANXA1 KO EVs maintained intermediate effects compared to MIA PaCa2 ones, here defined WT EVs. In detail, confocal analysis (panel A) described that when EVs are free of ANXA1 induced in a very weak way the protein translocation to the plasma membrane and only in rapid times (white arrows in panels b and e for 2 and 4 h of treatments). Thus, in this case, the effects of Gege3 appeared not strongly impactful (panels c, f, i, l). Furthermore, in all the functional assays ANXA1 KO EVs, less active than WT counterpart, appeared less influenced with respect to WT EVs by Gege3. This action is revealed in calcium mobilization (panel B), migration and invasion (panel C and D, respectively), and capillary-like structure formation (panel E).

Among the molecular aspects involved as a consequence of calcium mobilization, we used the molecules PMA and Gö6976 as activators and inhibitors of the protein PKCα, respectively [27,28]. We confirmed that the phosphorylation status of PKCα has been inhibited by Gö6976. This molecule has been also able to interfere with the positive effects of PMA. Interestingly, Gege3 acted in a very similar way to Gö6976, both alone and together with PMA (Figure 5C). Together with the different degrees of pPKCα, we also found that Gege3 blocked the activation by phosphorylation of ERK protein, which is known to be one of the effectors of PKCα. Indeed, the western blotting in Figure 5B showed that the pERK signal decreased in presence of Gege3 and of Gö6976, inversely it increased when HUVEC cells have been treated with PMA. Finally, ERK phosphorylation status returned to intermediate levels when Gege3 and Gö6976 have been added to PMA. The densitometry analysis, which better explained the western blotting image, has been included in Supplementary Figure S1B. By this analysis, we could clarify the significant effects of Gege3 on the phosphorylation status of PKCα and ERK and also of PMA and Gö6976, which we used as controls. 

Furthermore, through confocal analysis of HUVEC cells, we found that PMA, as an activator of PKCα, induced the translocation of ANXA1 to the plasma membrane as early as from 2 to 24 h (Figure 5D, panels b, g, l, q, white arrows) [29,30]. This rapid effect has been strongly reverted by Gö6976 if administered alone, in much the same way that we have witnessed in the case of Gege3 (see above in Figure 2C) but also in presence of PMA (Figure 5D, panels c, h, m, r for Gö6976 alone and panels d, i, n, s for Gö6976 and PMA, respectively). Therefore, the most interesting finding has been that also Gege3 has been able to inhibit the ANXA1 translocation to plasma membrane induced by PMA (Figure 5D, panels e, j, o, t). All these results have been further corroborated by fluorescence intensity analysis reported in Supplementary Figure S2C. 

Additionally, in Figure 5E we show the results obtained by cytofluorimetry assay which confirmed the different levels of HUVEC surface localization of ANXA1 after 2 and 16 h from treatments, selected as representative experimental points. Particularly, the green line represented the increased signal related to ANXA1 on the cell membrane following the PMA treatment, conversely, the pink one indicated the decreased presence of protein in this compartment in presence of Gö6976. In confirmation of what we have found by immunofluorescence, when PMA has been administered to HUVEC cells together with Gö6976 or Gege3, the signal related to ANXA1 became very similar to that of the untreated cells (orange and blue lines for PMA with Gö6976 and Gege3, respectively). For both experimental times, we confirmed the cytometry analysis by the quantitative evaluation performed on cell events positive for ANXA1 membrane staining. These results have been included in Supplementary Figure S3C. 

Finally, western blot analysis on compartmentalized protein extracts of total, cytosol, and membrane HUVEC cells treated for 8 h as here described, confirmed again the different degrees of localization to the plasma membrane of ANXA1 (Figure 5F). Thus, the protein movement induced by PMA has been notably inhibited by Gege3 and Gö6976. The densitometry analysis has been reported in Supplementary Figure S4E. 

### 3.6. Gege3 Interferes with the Effects on PKCα of Ac2-26 and MIA PaCa-2 EVs and with the Following Translocation of ANXA1 to Membrane

**Figure 6:**
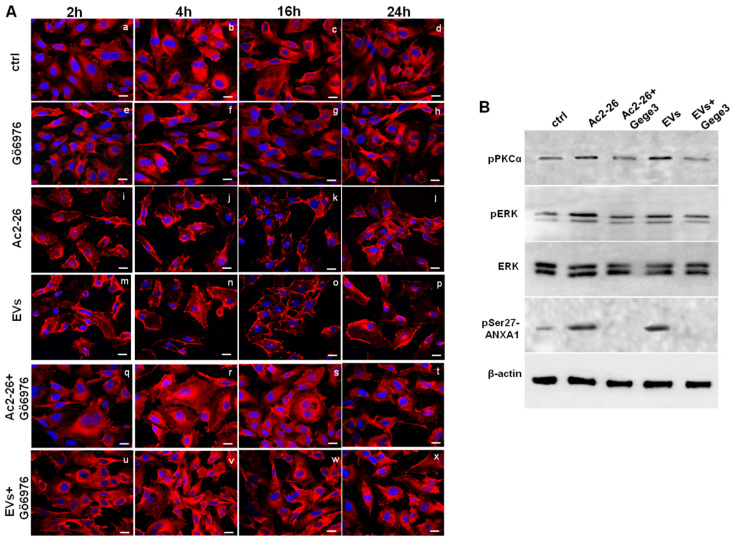
The interference of Gege3 on Ac2-26 and PC EVs affects HUVEC cells. (**A**) Immunofluorescence analysis to detect ANXA1 intracellular localization after treatments with Gö6976 (panels **e**–**h**), Ac2-26 (panels **i**–**l**), EVs (panels **m**–**p**), Ac2-26+Gö6976 (panels **q**–**t**) and EVs+Gö6976 (panels **u**–**x**) for 2, 4, 16 and 24 h, not treated cells (panels **a**–**d**). Magnification 63 × 1.4 NA. Bar = 100 μm. The data are representative of three independent experiments with similar results. (**B**) Western blotting for pPKCα, pERK, ERK, pSer27-ANXA1 normalized with β-actin on HUVEC cells treated with Ac2-26 1 µM, Ac2-26+Gege3, EVs and EVs+Gege3 for 15 min. Cropped blots from full-length gels are representative of three independent experiments with similar results.

In order to partly explain the mechanism by which Gege3 indirectly inhibits the translocation of ANXA1 to the plasma membrane of endothelial cells, we used the compound Gö6976 together with Ac2-26 or MIA PaCa-2 EVs to detect the protein intracellular localization. In Figure 6A, as for Gege3 co-treatment, we found that from 2 to 24 h of treatments, ANXA1 retained a cytosol diffuse localization in presence of the PKCα inhibitor Gö6976 (Figure 6A, panels q–t for Ac2-26+Gö6976 and panels u-x for Evs+Gö6976, respectively), unlike Ac2-26 and EVs taken as a single treatment (Figure 6A, panels h–l for Ac2-26 and panels m–p for EVs, respectively). Also in this case, as reported in Supplementary Figure S2D, we confirmed the confocal analysis by the evaluation of the CMCF (Corrected Membrane Cell Fluorescence). 

Finally, we focused again on PKCα involvement finding that Ac2-26 and EVs induced a significant increase in the phosphorylation status of this kinase. Once again, the assessment of PKCα activation has been further supported by the increase of phospho-ERK following the treatments of ANXA1 mimetic peptide and PC-derived microvesicles. Interestingly, the phosphorylation of both kinds of kinases underwent a strong inhibition when Ac2-26 and EVs have been added to HUVEC cells together with Gege3. This notable variation has been revealed in the western blotting of Figure 6B and confirmed by densitometry analysis reported in Supplementary Figure S1C. Furthermore, it is known that the phosphorylation on the ANXA1 serine 27 residue is the crucial post-translational modification to induce the protein translocation to the plasma membrane. Moreover, this modification is closely related to PKCα activation [7]. For these reasons, we investigated the phospho-ser27-ANXA1 expression, assessing its appearance in presence of Ac2-26 and PC EVs. When these treatments have been associated with Gege3, this post-translational modification disappeared, according to the inhibition of PKCα (Figure 6B).

## 4. Discussion

The promising antiangiogenic activity of the compound Gege3 has been studied in in vitro and in vivo systems highlighting the ability of this molecule to interfere with the activation of several kinases as ERK, Akt, and dystrophia myotonica protein kinase (DMPK)1, known to be some of the intermediate elements of VEGF signaling in endothelial cells. Interestingly, the effects of Gege3 have been explained through the inhibition of the calcium intracellular mobilization induced by the interaction that Gege3 generates with the protein calreticulin [14,15]. In this study, we have investigated one of the mechanisms by which Gege3 can interfere with protein elements involved in the angiogenesis process in a calcium-mediated fashion. 

We have focused on ANXA1 since it is a protein known to retain an important role in the activation of endothelial cells mainly in the PC microenvironment. Our studies have shown in-depth that the extracellular form of this protein, as the content of PC-derived EVs, induces the activation of tumor cells as an autocrine element and the malignant transformation of the microenvironment as a paracrine actor [9,10]. Moreover, it has been observed in depth that the ANXA1 behavior is closely influenced by calcium intracellular trafficking [8,31]. 

Here, the potential opposite correlation Gege3/ANXA1 has been proved by the Gege3-dependent inhibition of endothelial cell migration/invasion, angiogenesis process, and calcium mobilization induced by Ac2-26. 

The intracellular localization of ANXA1 represents an essential concern in ensuring the protein effects. In particular, when associated with the plasma membrane, ANXA1 can be accessible to receptor partners, mainly FPRs, triggering oncogenic pathways [32]. Furthermore, it has been shown that the ANXA1 mimetic peptide is able to induce a positive loop enhancing the protein translocation to the plasma membrane and its following externalization mainly as the content of microvesicles [33]. Moreover, we have proved, for the first time, that this positive loop is induced by the PC-derived EVs on endothelial cells. This paracrine effect can be probably due to the presence of ANXA1 on the surface of these microvesicles. 

Our finding that Gege3 is able to inhibit these induced effects of the extracellular form of ANXA1 suggested that this molecule could significantly interfere with the cross-talk that ANXA1 performs in the tumor microenvironment to promote PC progression. In fact, the inhibition by Gege3 of all the functional aspects evaluated in terms of EVs-induced endothelial cells activation as migration/invasion, angiogenesis, and calcium release represents an appealing confirmation of this cue. It is important to note that the effects induced by EVs on the translocation of ANXA1 to the plasma membrane are not completely inhibited by Gege3. This aspect could also be related to other factors involved in the activation of EVs. It may not require calcium mobilization or ANXA1 activity. In any case, our data allowed us to speculate that Gege3 can also have a role in the vesiculation process probably through indirect blocking of the translocation of ANXA1 to the plasma membrane. Actually, the specific involvement of ANXA1 in this process is not yet established, nevertheless, an increasing number of experimental pieces of evidence places this protein as one of the major players in this process [9,33]. Indeed, the post-translational modifications and the binding to Ca^2+^ have been described as important features in regulating the association of ANXA1 to vesicles [34,35]. Thus, it is conceivable that Gege3 could represent an interesting means by which to investigate how ANXA1 specifically intervenes in the production or even in the externalization of microvesicles.

The relevance of the interference by Gege3 on the ANXA1 behavior has found a further confirmation using of EVs from ANXA1 KO MIA PaCa-2 cells. These microvesicles, overall less active of WT EVs counterpart on the activation of HUVEC receiving cells, were partially blocked by Gege3. The assessment of this moderate negative influence suggests that additional mechanisms could be involved.

Nevertheless, in order to support our assumptions, we proved the molecular mechanism by which ANXA1 does not move to the plasma membrane in presence of Gege3. Interestingly, the signaling we assessed has revealed that the strong decrease of calcium intracellular amount leads to the inhibition of calcium-dependent PKCα which, in turn, is not more able to promote the phosphorylation of ANXA1 on the residue of serine 27. The relevance of this modification is also highlighted by the presence of phospho-ANXA1 in EVs secreted by MIA PaCa-2 strongly suggesting again that once the serine 27 is phosphorylated ANXA1 is led to be externalized through vesiculation in PC. Thus, since PKCα has been not found as directly affected by Gege3 [15], we hypothesized that this effect is mediated indirectly. It is known that this modification is crucial for the translocation to the plasma membrane of ANXA1 and, in this cell compartment, the protein can explicate the great part of its activities, as stated above [31,36]. Thus, the inhibition of ser27 phosphorylation blocks this signaling indirectly preventing some of the downstream oncogenic ANXA1 effects in the PC progression [18,37]. This clue has allowed us to speculate that the investigation of the potential impact of the hindrance to serine 27 phosphorylation can represent an appealing challenge to assess the ANXA1 behavior. In this way, we will propose to designate the mechanism of protein externalization, particularly thanks to vesicles, and later of its uptake, in the cross-talk which the primary tumor builds with the EVs receiving cell elements in the microenvironment. Different kinds of technical approaches could be used, in addition, or not to Gege3, in order to find some aspects of these mechanisms. 

Taken together, our data encourage future investigation about the molecular aspects through which Gege3 acts as an antiangiogenic compound in a contest of tumor development. The finding of the involvement of ANXA1 in these effects surely makes this molecule even more attractive in the scenario of the research of different potential antitumor agents [38].

## 5. Conclusions

The pyrazolyl-urea molecule Gege3 has been found able to inhibit the angiogenesis correlated to PC development, as shown in other tumor models [13,15]. This effect has been revealed in this study about the activation of endothelial cells promoted by cancer-derived microvesicles. Thus, Gege3 has turned out as an interfering element in a finely regulated cross-talk among PC cells and the tumor microenvironment in which one of the major actors is represented by ANXA1 protein. In particular, we showed that the inhibition of calcium mobilization in endothelial cells, induced by Gege3, prevented the translocation of ANXA1 to the plasma membrane promoted by the extracellular counterpart of this protein in paracrine positive feedback. Therefore, Gege3 indirectly inhibits the PKCα activation and its downstream events which are known to trigger the phosphorylation of ANXA1 on serine 27, a post-translational modification leading to the movement of the protein to plasma membrane inducing oncogenic effects. All these findings have allowed us to include Gege3 in a library of potential antitumor agents, interestingly worthy of further investigation. In particular, it would be attractive studying a direct correlation between Gege3 and the inhibition of PC progression first on tumor cells and later in in vivo models.

## Data Availability

The data presented in this study are available upon request from the corresponding author.

## Supplementary Materials

The following are available online at https://www.mdpi.com/article/10.3390/biom11121758/s1, Figure S1: EVs characterization and densitometry analyses; Figure S2: CMCF analyses; Figure S3: quantitative analyses of cell membrane ANXA1 by flow cytometry; Figure S4: densitometry analyses of western blotting of compartimentalized protein extracts; Figure S5: effects of Gege3 on ANXA1 KO EVs activity.

## References

[1] Lugano R., Ramachandran M., Dimberg A. (2020). Tumor angiogenesis: Causes, consequences, challenges and opportunities. Cell Mol. Life Sci..

[2] Carmeliet P., Jain R.K. (2011). Molecular mechpanisms and clinical applications of angiogenesis. Nature.

[3] Jiang X., Wang J., Deng X., Xiong F., Zhang S., Gong Z., Li X., Cao K., Deng H., He Y. (2020). The role of microenvironment in tumor angiogenesis. J. Exp. Clin. Cancer Res..

[4] Foo S.L., Yap G., Cui J., Lim L.H.K. (2019). Annexin-A1—A Blessing or a Curse in Cancer?. Trends Mol. Med..

[5] Guo C., Liu S., Sun M.Z. (2013). Potential role of Anxa1 in cancer. Future Oncol..

[6] Fu Z., Zhang S., Wang B., Huang W., Zheng L., Cheng A. (2020). Annexin A1: A double-edged sword as novel cancer biomarker. Clin. Chim. Acta.

[7] D’Acunto C.W., Gbelcova H., Festa M., Ruml T. (2014). The complex understanding of Annexin A1 phosphorylation. Cell. Signal..

[8] Belvedere R., Novizio N., Pessolano E., Tosco A., Eletto D., Porta A., Campiglia P., Perretti M., Filippelli A., Antonello Petrella A. (2020). Heparan sulfate binds the extracellular Annexin A1 and blocks its effects on pancreatic cancer cells. Biochem. Pharmacol..

[9] Pessolano E., Belvedere R., Bizzarro V., Franco P., De Marco I., Porta A., Tosco A., Parente L., Perretti M., Petrella A. (2018). Annexin A1 May Induce Pancreatic Cancer Progression as a Key Player of Extracellular Vesicles Effects as Evidenced in the In Vitro MIA PaCa-2 Model System. Int. J. Mol. Sci..

[10] Novizio N., Belvedere R., Pessolano E., Tosco A., Porta A., Perretti M., Campiglia P., Filippelli A., Petrella A. (2020). Annexin A1 Released in Extracellular Vesicles by Pancreatic Cancer Cells Activates Components of the Tumor Microenvironment, through Interaction with the Formyl-Peptide Receptors. Cells.

[11] Pessolano E., Belvedere R., Novizio N., Filippelli A., Perretti M., Whiteford J., Petrella A. (2021). Mesoglycan connects Syndecan-4 and VEGFR2 through Annexin A1 and formyl peptide receptors to promote angiogenesis in vitro. FEBS J..

[12] Abdalla A.M.E., Xiao L., Ullah M.W., Yu M., Ouyang C., Yang G. (2018). Current Challenges of Cancer Anti-angiogenic Therapy and the Promise of Nanotherapeutics. Theranostics.

[13] Meta E., Brullo C., Sidibe A., Imhof B.A., Bruno O. (2017). Design, synthesis and biological evaluation of new pyrazolyl-ureas and imidazopyrazolecarboxamides able to interfere with MAPK and PI3K upstream signaling involved in the angiogenesis. Eur. J. Med. Chem..

[14] Morretta E., Belvedere R., Petrella A., Spallarossa A., Rapetti F., Bruno O., Brullo C., Monti M.C. (2021). Novel insights on the molecular mechanism of action of the anti-angiogenic pyrazolyl-urea GeGe-3 by functional proteomics. Bioorg. Chem..

[15] Meta E., Imhof B.A., Ropraz P., Fish R.J., Brullo C., Bruno O., Sidibé A. (2017). The pyrazolyl-urea GeGe3 inhibits tumor angiogenesis and reveals dystrophia myotonica protein kinase (DMPK)1 as a novel angiogenesis target. Oncotarget.

[16] Belvedere R., Bizzarro V., Parente L., Petrella F., Petrella A. (2017). The Pharmaceutical Device Prisma^®^ Skin Promotes in Vitro Angiogenesis through Endothelial to Mesenchymal Transition during Skin Wound Healing. Int. J. Mol. Sci..

[17] Belvedere R., Bizzarro V., Forte G., Dal Piaz F., Parente L., Petrella A. (2016). Annexin A1 contributes to pancreatic cancer cell phenotype, behaviour and metastatic potential independently of Formyl Peptide Receptor pathway. Sci. Rep..

[18] Bizzarro V., Fontanella B., Carratù A., Belvedere R., Marfella R., Parente L., Petrella A. (2012). Annexin A1 N-terminal derived peptide Ac2-26 stimulates fibroblast migration in high glucose conditions. PLoS ONE.

[19] Belvedere R., Saggese P., Pessolano E., Memoli D., Bizzarro V., Rizzo F., Parente L., Weisz A., Petrella A. (2018). miR-196a Is Able to Restore the Aggressive Phenotype of Annexin A1 Knock-Out in Pancreatic Cancer Cells by CRISPR/Cas9 Genome Editing. Int. J. Mol. Sci..

[20] Belvedere R., Morretta E., Pessolano E., Novizio N., Tosco A., Porta A., Whiteford J., Perretti M., Filippelli A., Monti M.C. (2021). Mesoglycan exerts its fibrinolytic effect through the activation of annexin A2. J. Cell Physiol..

[21] Belvedere R., Pessolano E., Porta A., Tosco A., Parente L., Petrella F., Perretti M., Petrella A. (2020). Mesoglycan induces the secretion of microvesicles by keratinocytes able to activate human fibroblasts and endothelial cells: A novel mechanism in skin wound healing. Eur. J. Pharmacol..

[22] Bizzarro V., Belvedere R., Pessolano E., Parente L., Petrella F., Perretti M., Petrella A. (2019). Mesoglycan induces keratinocyte activation by triggering syndecan-4 pathway and the formation of the annexin A1/S100A11 complex. J. Cell Physiol..

[23] D’Acunto W.C., Fontanella B., Rodriquez M., Taddei M., Parente L., Petrella A. (2010). Histone deacetylase inhibitor FR235222 sensitizes human prostate adenocarcinoma cells to apoptosis through up-regulation of Annexin A1. Cancer Lett..

[24] Solito E., Mulla A., Morris J.F., Christian H.C., Flower R.J., Buckingham J.C. (2003). Dexamethasone induces rapid serine-phosphorylation and membrane translocation of annexin 1 in a human folliculostellate cell line via a novel nongenomic mechanism involving the glucocorticoid receptor, protein kinase C, phosphatidylinositol 3-kinase, and mitogen-activated protein kinase. Endocrinology.

[25] Yi M., Schnitzer J.E. (2009). Impaired tumor growth, metastasis, angiogenesis and wound healing in annexin A1-null mice. Proc. Natl. Acad. Sci. USA.

[26] Pin A.L., Houle F., Fournier P., Guillonneau M.M., Paquet E.R., Simard M.J., Royal I., Huot J. (2012). Annexin-1-mediated Endothelial Cell Migration and Angiogenesis Are Regulated by Vascular Endothelial Growth Factor (VEGF)-induced Inhibition of miR-196a Expression. J. Biol. Chem..

[27] Gao Q., Tan J., Ma P., Ge J., Liu Y., Sun X., Zhou L. (2009). PKC alpha affects cell cycle progression and proliferation in human RPE cells through the downregulation of p27kip1. Mol. Vis..

[28] Masur K., Lang K., Niggemann B., Zanker K.S., Entschladen F. (2001). High PKC alpha and low E-cadherin expression contribute to high migratory activity of colon carcinoma cells. Mol. Biol. Cell.

[29] Solito E., Christian H.C., Festa M., Mulla A., Tierney T., Flower R.J., Buckingham J.C. (2006). Post-translational modification plays an essential role in the translocation of annexin A1 from the cytoplasm to the cell surface. FASEB J..

[30] Rosengarth A., Luecke H. (2003). A calcium-driven conformational switch of the N-terminal and core domains of annexin A1. J. Mol. Biol..

[31] Boudhraa Z., Bouchon B., Viallard C., D’Incan M., Degoul F. (2016). Annexin A1 localization and its relevance to cancer. Clin. Sci. (Lond.).

[32] Boye T.L., Jeppesen J.C., Maeda K., Pezeshkian W., Solovyeva V., Nylandsted J., Simonsen A.C. (2018). Annexins induce curvature on free-edge membranes displaying distinct morphologies. Sci. Rep..

[33] Pessolano E., Belvedere R., Bizzarro V., Franco P., De Marco I., Petrella F., Porta A., Tosco A., Parente L., Perretti M. (2019). Annexin A1 Contained in Extracellular Vesicles Promotes the Activation of Keratinocytes by Mesoglycan Effects: An Autocrine Loop Through FPRs. Cells.

[34] Eden E.R., Sanchez-Heras E., Tsapara A., Sobota A., Levine T.P., Futter C.E. (2016). Annexin A1 Tethers Membrane Contact Sites that Mediate ER to Endosome Cholesterol Transport. Dev. Cell.

[35] Rogers M.A., Buffolo F., Schlotter F., Atkins S.K., Lee L.H., Halu A., Blaser M.C., Tsolaki E., Higashi H., Luther K. (2020). Annexin A1-dependent tethering promotes extracellular vesicle aggregation revealed with single-extracellular vesicle analysis. Sci. Adv..

[36] Bizzarro V., Belvedere R., Dal Piaz F., Parente L., Petrella A. (2012). Annexin A1 induces skeletal muscle cell migration acting through formyl peptide receptors. PLoS ONE.

[37] Belvedere R., Bizzarro V., Popolo A., Dal Piaz F., Vasaturo M., Picardi P., Parente L., Petrella A. (2014). Role of intracellular and extracellular annexin A1 in migration and invasion of human pancreatic carcinoma cells. BMC Cancer.

[38] Petrella A., Fontanella B., Carratù A., Bizzarro V., Rodriquez M., Parente L. (2011). Histone deacetylase inhibitors in the treatment of hematological malignancies. Mini Rev. Med. Chem..

