# Supplementary material for: The Pyrazolyl-Urea Gege3 Inhibits the Activity of ANXA1 in the Angiogenesis Induced by the Pancreatic Cancer Derived EVs
Source: Biomolecules. 2021 Nov 24;11(12):1758. doi: 10.3390/biom11121758 (PMC8699007; doi:10.3390/biom11121758)
Supplement: Supplementary file 1 [file biomolecules-11-01758-s001.zip › biomolecules-1432709-Supplementary.pdf]

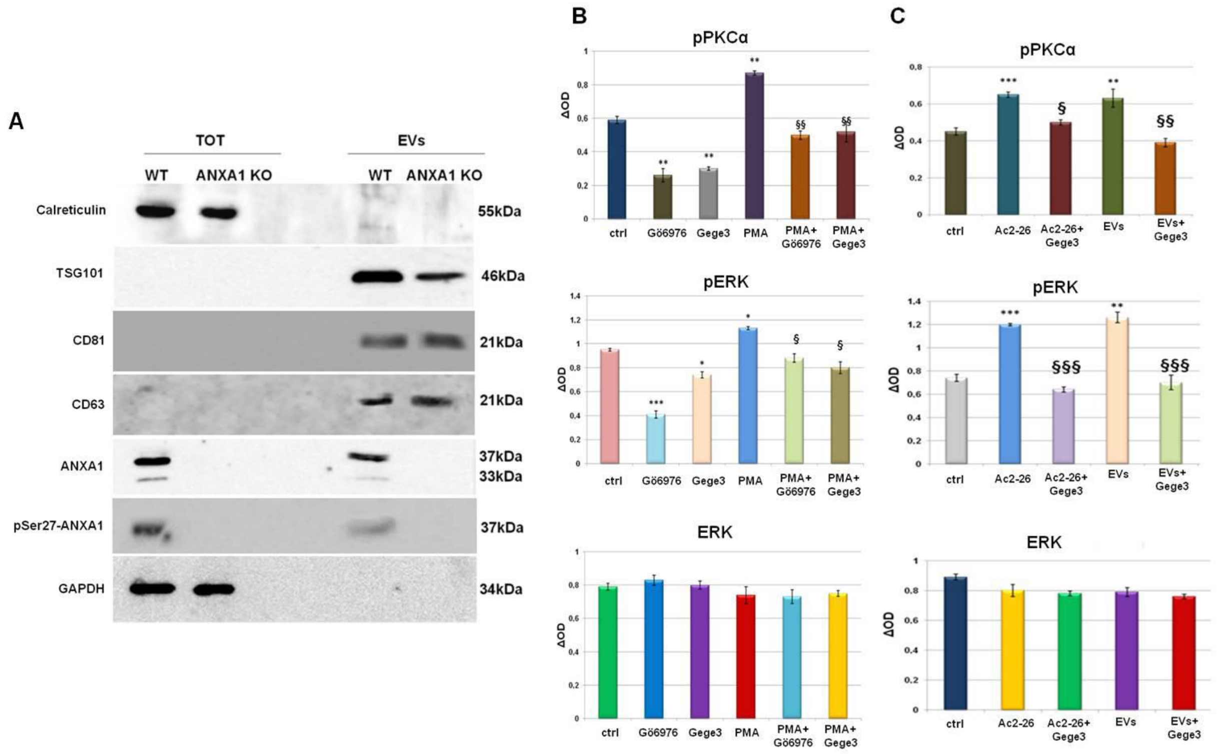

Supplementary Figure S1. （A）Western blot analysis of protein extracts from MIA PaCa-2 cell and EVs. Crpped blots from full-length gels are representative of three independent experiments with similar results using antibodies against calreticulin, TSG101, CD81, CD63, ANXA1, pSer27-ANXA1 and normalized with GAPDH. Densitometry analysis of western blotting on total protein extracts from HUVEC cells treated or not for 15 minutes with (B) PMA 100nM, Gege3 10μM, Gö6976 1μM, PMA+Gö6976 and with (C) Ac2-26 1μM, Ac2-26+Gege3, EVs and EVs+Gege3. The protein signal assessed have been pPKCα, pERK and ERK normalized on β-action one. * $p<0.05$; ** $p<0.01$; *** $p<0.001$ for treated cells vs untreated controls; § $p<0.05$; §§ $p<0.01$; §§§ $p<0.001$ for Gege3 and Gö6976 treated cells vs. the positive activity of PMA, Ac2-26 and EVs.

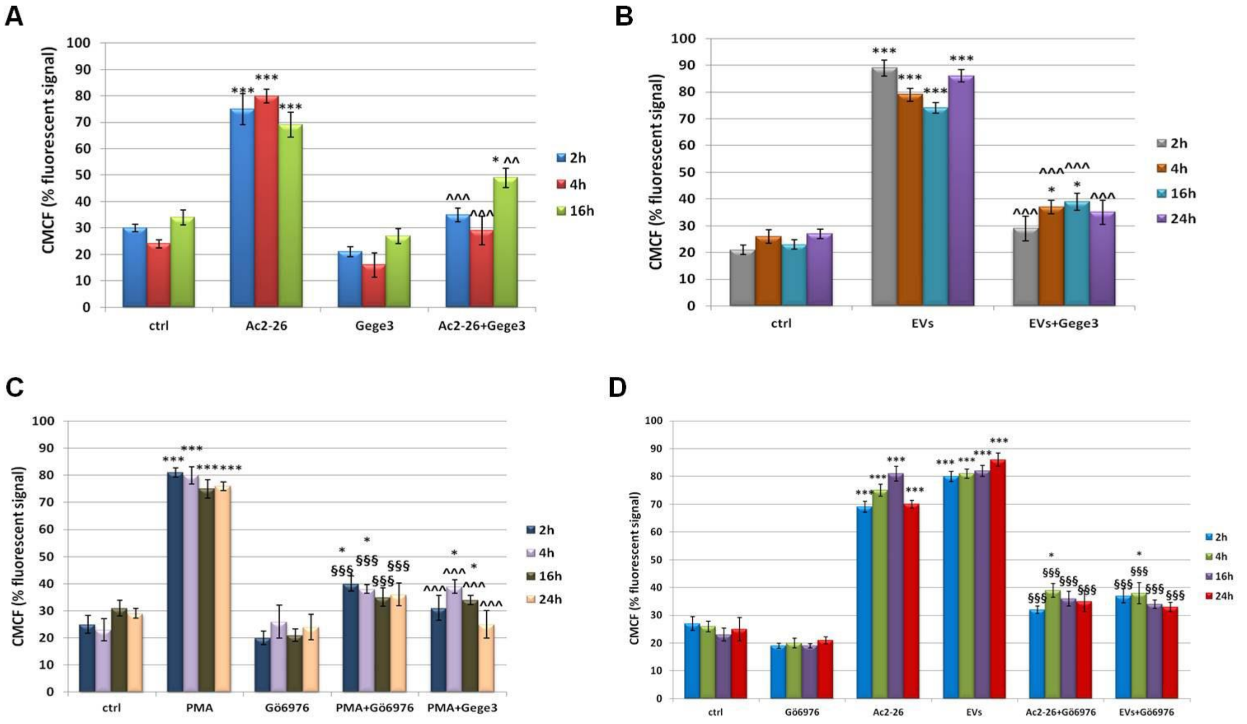

Supplementary Figure S2. CMCF (Corrected Membrane Cell Fluorescence) has been calculated as percentage of fluorescent signal as reported in Mate1ial and methods section. The histogram revealed the ANXAl signal on plasma membrane of HUVEC cells after treatments with (A) Ac2-26 lμM. Gege3 10μM and both of them; (B) EVs with and without Gege3 10μM; (C) PMA l00nM. Gö6976 lμM, PMA+Gö6976. PMA+Gege3; (D) Ac2-26+Gö6976 and EVs+Gö6976.*p<0.05; ***p<0.001 for treated cells vs. untreated controls; ^p<0.05; ^^^p<0.001 for cells treated with Ac2-26 and Gege3/PMA vs. Gege3 alone;§§§ p<0.001 for PMA+Gö6976; Ac2-26+Gö6976 and EVs+Gö6976 treatments vs. Gö6976 alone

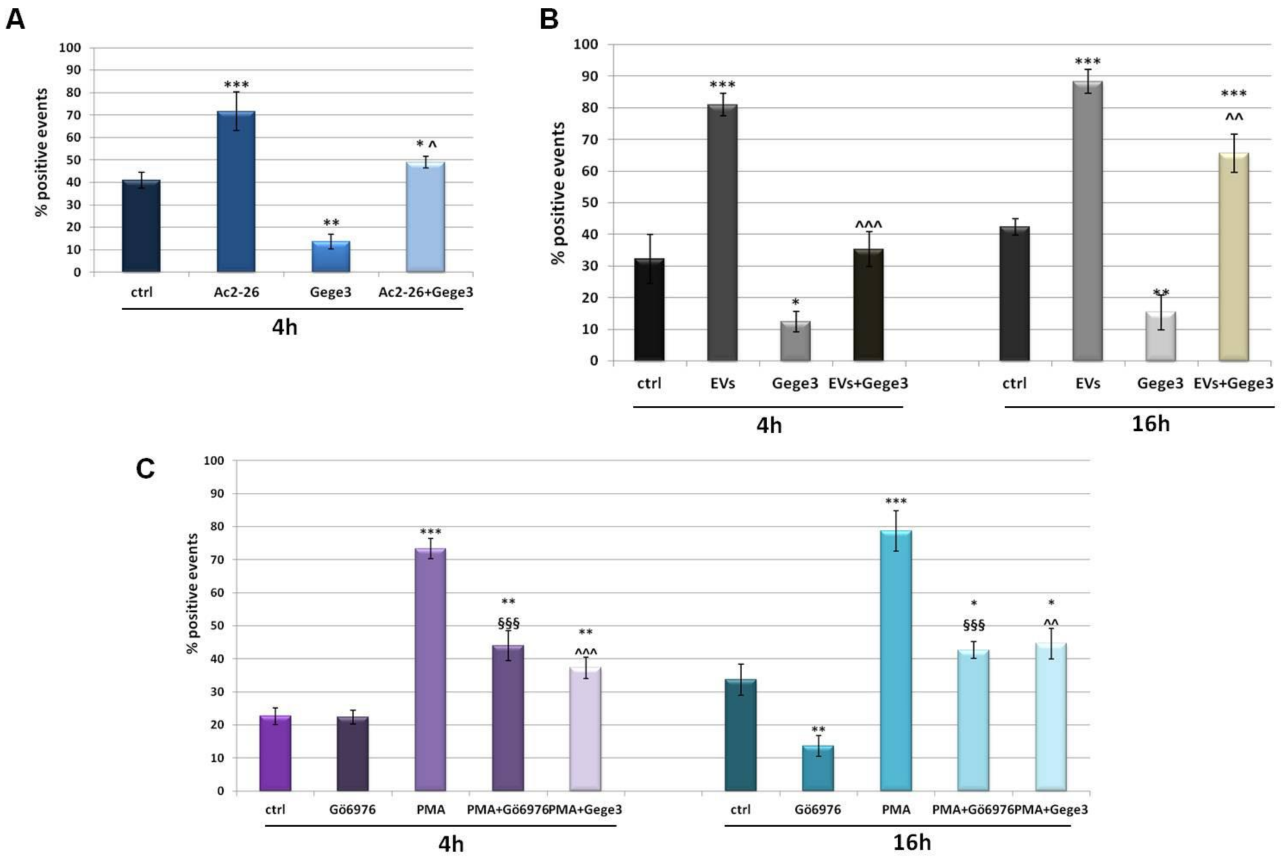

Supplementary Figure S3. Quantitative analysis of flow cytometry evaluation of ANXAl on HUVEC plasma membrane. Related to the graphs reported in figure 2C (A); figure 3B (B); figure 5D (D).*p<0.05; **p<0.01 ***p<0.001 for treated cells vs. untreated controls; ^p<0.05 ^^p<0.01;^^^p<0.001 for cells treated with Ac2-26/PMA and Gege3 vs. Gege3 alone; §§§p<0.001 for PMA+Gö6976 treatment vs. Gö6976 alone

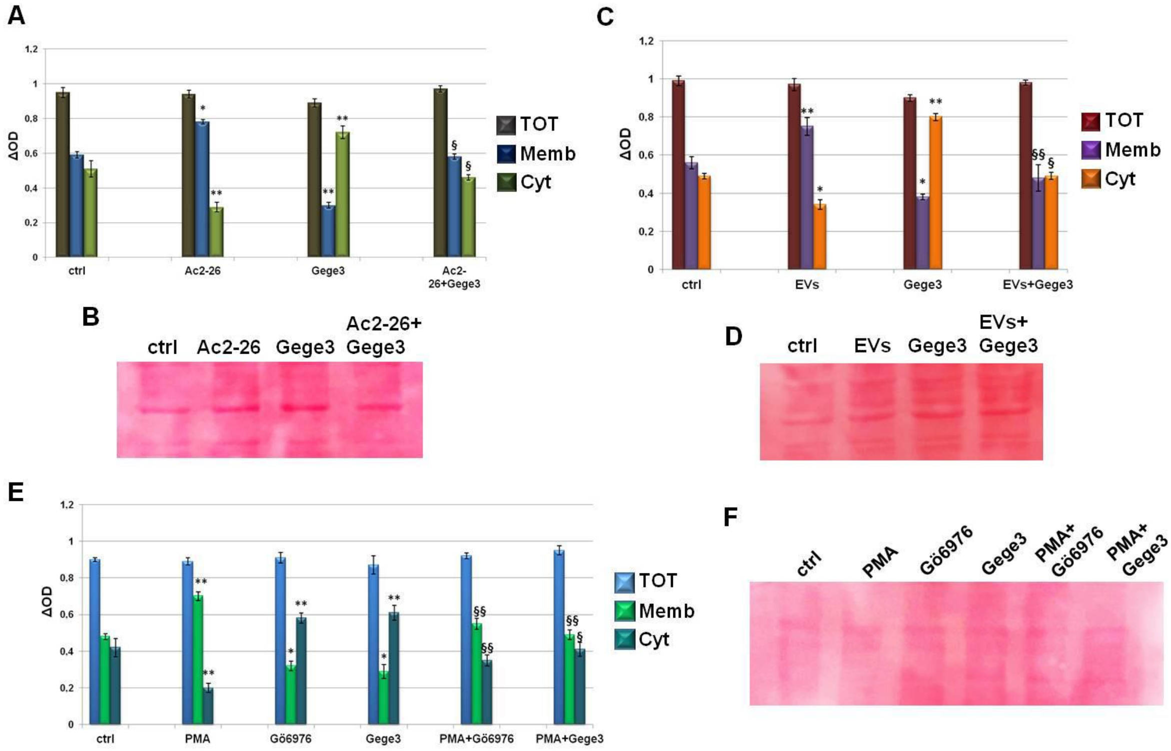

Supplementary Figure S4. Densitometry analysis of western blotting on total, cytosol and membrane protein extracts from HUVEC cells treated or not for 8 h (A) with Ac2-26 1μM, Gege3 l0μM, Ac2-26+Gege3; (C) with MIA PaCa-2 EVs, Gege3 l0μM and EVs+Gege3; (E) PMA l00nM, Gege3 l0μM, Gö6976 lμM, PMA+Gö6976, PMA+Gege3. The total and cytosol ANXAl signal assessed has been normalized on β-actin one. The ANXAl membrane signal has been analyzed on the related red poncaeu shown in (B), (D) and (F).*$p<0.05$;**$p<0.01$ for treated cells vs. untreated controls; § $p<0.05$; §§ $p<0.01$ for Gege3 and Gö6976 treated cells vs. the positive activity of PMA, Ac2-26 and EVs

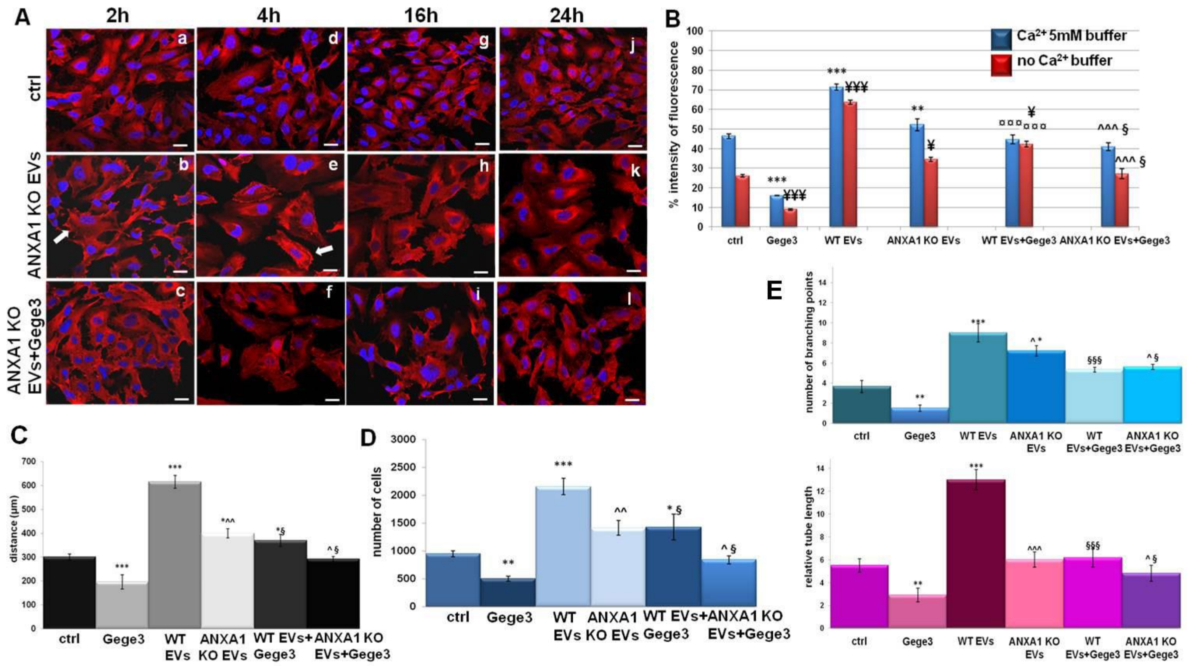

Supplementary Figure S5. (A) Confocal analysis for ANXA1 on cells treated or not with ANXAl KO EVs with and without Gege.3 l0μM for 2(panels a-c), 4 (panels d-f), 16 (panels g-i) and 24 (panels j-l) hours. Magnification 63×1.4 NA Bar=100 μM. (B) Effects of WT and ANXA1 KO EVs with and without Gege3 on calcium mobilization in presence of $CaCl_2$, 5mM (blue bars) or not (red ones). (C) in vitro angiogenesis evaluated by number of branching points and relative tube length after 12 hours of treatments. Migration (D) and invasion (E) analyses in presence of the same experimental points after 24hours of treatment. Data are means ± standard deviation of three experiments with similar results. *p<0.05: ; **p<0.01; ***p<0.001 for treated cells vs. non treated controls (in presence of calcium in case of Fluo-4am assay); ¤¤¤ p<0.001 for WT EVs+Gege3 vsWT EVs alone both in presence and non of Ca^{2+} ; ¥p<0.05; ¥¥¥p<0.001 for treated cells vs. non treated control in buffer without Ca^{2+} § p<0.05; §§§ p<0.001 for WT and ANXA1 KO EVs treated cells vs. WT andANXA1 KO EVs with Gege3; ^ p<0.05; ^^p<0.01 for ANXAl KO EVs(±Gege3) vs. WT EVs(±Gege3)
